# An Improved Nested U-Net Network for Fluorescence In Situ Hybridization Cell Image Segmentation

**DOI:** 10.3390/s24030928

**Published:** 2024-01-31

**Authors:** Zini Jian, Tianxiang Song, Zhihui Zhang, Zhao Ai, Heng Zhao, Man Tang, Kan Liu

**Affiliations:** School of Electronic and Electrical Engineering, Wuhan Textile University, Wuhan 430200, China; znjian@wtu.edu.cn (Z.J.); 2215363012@mail.wtu.edu.cn (T.S.); 2315363051@mail.wtu.edu.cn (Z.Z.); 2016036@wtu.edu.cn (Z.A.); 1803240222@mail.wtu.edu.cn (H.Z.)

**Keywords:** FISH images, cell contours, segmentation, Unet++, attention

## Abstract

Fluorescence in situ hybridization (FISH) is a powerful cytogenetic method used to precisely detect and localize nucleic acid sequences. This technique is proving to be an invaluable tool in medical diagnostics and has made significant contributions to biology and the life sciences. However, the number of cells is large and the nucleic acid sequences are disorganized in the FISH images taken using the microscope. Processing and analyzing images is a time-consuming and laborious task for researchers, as it can easily tire the human eyes and lead to errors in judgment. In recent years, deep learning has made significant progress in the field of medical imaging, especially the successful application of introducing the attention mechanism. The attention mechanism, as a key component of deep learning, improves the understanding and interpretation of medical images by giving different weights to different regions of the image, enabling the model to focus more on important features. To address the challenges in FISH image analysis, we combined medical imaging with deep learning to develop the SEAM-Unet++ automated cell contour segmentation algorithm with integrated attention mechanism. The significant advantage of this algorithm is that it improves the accuracy of cell contours in FISH images. Experiments have demonstrated that by introducing the attention mechanism, our method is able to segment cells that are adherent to each other more efficiently.

## 1. Introduction

Fluorescence in situ hybridization (FISH) allows for the identification and detection of precise DNA sequences or RNA molecules within cells or tissue samples via staining [1,2]. This technique is highly effective in providing researchers with chromosomal and genomic information that aids in the comprehension of gene rearrangements, cytogenetic variation, as well as chromosomal abnormalities and localization. FISH technology finds wide application in medical diagnosis, including tumor diagnosis, genetic disease screening, and embryo genome analysis. Its potential in the early diagnosis, prognosis, and treatment of leukemia, cancer, Down syndrome, and other conditions is significant [3,4,5]. This technique enables the quantification and localization of specific genes providing crucial information for disease diagnosis and treatment. In recent years, the integration of medical imaging and computer science has become increasingly close. Medical image detection methods using deep learning have become a popular research topic [6,7,8,9,10,11]. However, medical image data are often large and complicated, and there are only limited labeled data available, which increases the difficulty of training deep learning models. Furthermore, FISH images contain sensitive patient information, making dataset acquisition even more challenging.

Segmentation in medical images requires higher accuracy compared to natural images. In natural images, precise segmentation masks may not hold utmost importance. However, in medical imagery, even minor segmentation errors can result in inaccurate clinical outcomes. For instance, ensuring precise segmentation of cell outlines in FISH cell images is crucial for later individual cell analysis. In an experimental setting, cells are usually clustered together. To accurately characterize the morphology, size, and location of individual cells, it is essential to precisely segment their outlines beforehand. Minimal segmentation errors can cause experimental results to be inaccurate impacting the pathological comprehension of FISH images. Traditional segmentation algorithms exhibit inaccuracies and unreliability when dealing with cells with low resolution and adherent contours. For example, threshold-based region segmentation or edge-based detection by practical operators such as Sobel or Laplacian operate according to simple rules [12]. Thresholds or discontinuous local features with precise grayscale information can be observed in these methods, especially in regions with high local contrast. That is, these methods rely heavily on the fundamental properties of the image. When there is more noise or discontinuous gray scale variation in the image, the segmentation results are not satisfactory. In order to solve the problem of imprecise segmentation of fine features, deep learning-based image segmentation techniques are essential.

Attention is a crucial and complex cognitive function for humans [13,14]. Humans do not process complete information simultaneously, but selectively focus on specific parts when necessary, while ignoring other perceptible information [15,16]. For instance, in visual perception, people usually do not observe the whole scene continuously, but focus on specific parts when needed. When individuals consistently identify a specific aspect of a scene as noteworthy, they develop the ability to concentrate on that aspect and prioritize relevant information when encountering similar scenes in the future. This method enables humans to efficiently and accurately select and focus on valuable information amidst a large amount of data [17,18]. Motivated by this observation, attention mechanisms were introduced into computer vision with the aim of imitating this aspect of the human visual system [19,20,21,22]. Such an attention mechanism can be regarded as a dynamic weight adjustment process based on features of the input image.

Convolutional neural networks (CNNs) are a widely used deep learning framework and have attracted great interest in the field of medical image analysis [23,24,25]. UNet is a convolutional neural network that was introduced by Olaf Ronneberger [26] in 2015 for the purpose of medical image segmentation. The network employs an encoder–decoder architecture along with jump connections. This combination can efficiently maintain and retrieve detailed information from characteristic maps, making it an ideal choice for medical imaging analysis with limited sample data. In 2018, Zongwei Zhou and extended UNet and introduced UNet++ (Nested Unet) [27]. The primary concept of UNet++ is to include numerous sub UNets within UNet’s decoder to enhance segmentation performance. Multiple feature representations are also introduced to enable the model to capture diverse details and scales of the target object. Many new models have been proposed to improve the accuracy of segmentation capability. In 2019, Jianhuo Shen et al. [28,29] proposed the Mini-u-net model to perform automatic detection of cell contours. In 2021, Li D et al. [30] proposed a new model by combining Neural Ordinary Differential Equations with Unet to segment cell contours. In 2022, Thi Le P et al. [31] used a CBA network to perform nucleus segmentation without manual intervention. Deep learning methods can be highly effective when there is a large number of samples available during the training phase [32]. However, in many medical applications, the images are much smaller, typically less than 1000. Therefore, one of the main challenges in applying deep learning to medical images is to train the model with a limited dataset without overfitting. To address this issue, various approaches have been proposed. For instance, some suggest using 2D or 3D image patches as input instead of full-size images [33,34,35,36], while others propose transfer learning by utilizing models trained on a large number of natural images in computer vision [37,38,39]. Due to the limited number of FISH images and the high requirements for segmentation accuracy, we propose the SEAM-Unet++ deep learning model to improve the accuracy of the model for FISH cell image segmentation by fusing the SEAM attention mechanism with Unet++.

The contributions of this paper are mainly:

1. An improved network, SEAM-Unet++, based on the UNet++ network is proposed for cell contour segmentation in FISH images.
2. Noting the weak cell boundaries, to further enhance the ability to extract valid features, we implement a convolutional block attention module (SEAM) in the UNet++ network to enhance the responsiveness of the output partially occluded contours and utilize depth-separable convolution to learn spatial dimensionality and channel correlation.

## 2. Materials 

### 2.1. Image Acquisition

**Figure 1:**
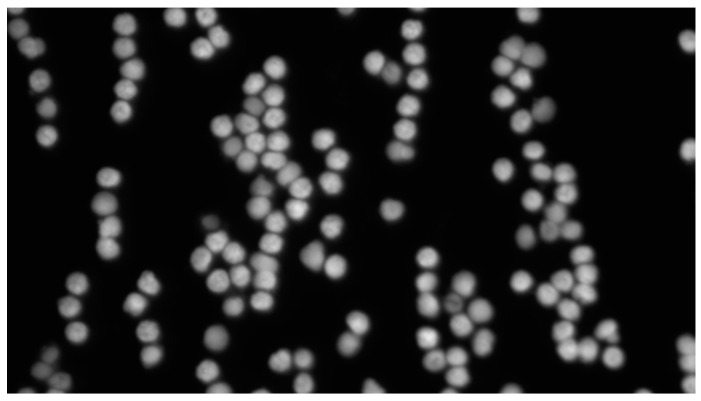
Example of datasets.

All FISH images used in this study were taken and provided by professional staff in our laboratory. The equipment used for image acquisition was an inverted fluorescence microscope with specification model IX83, and we collected a total of 246 cells images at 60× size. The representative images are shown in Figure 1.

### 2.2. Image Preprocessing

**Figure 2:**
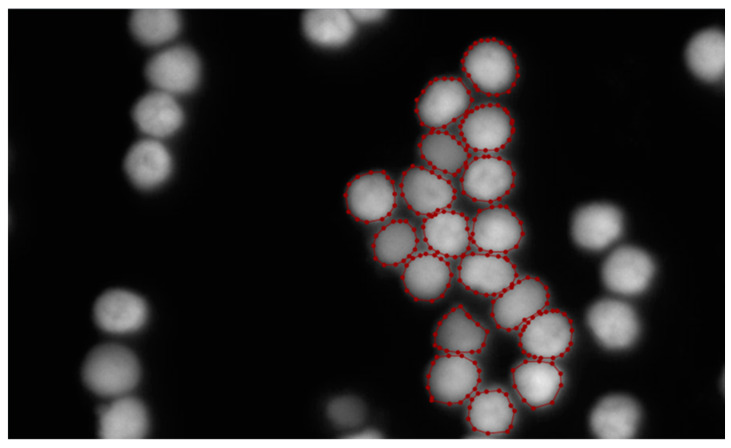
Labeling process.

We labelled 199 FISH cells images of size 1920 × 1080 using Labelme 5.2.1 software. The labelling process is depicted in Figure 2. The outline of the cell is outlined with a number of points to produce a final mask. The limited number of images necessitates the expansion of the dataset [40]. We apply various data augmentation techniques, including cropping, rotation, mirroring, raising gray value, and adding Gaussian noise to the original images. Cropping enhances the model’s adaptability to changes in object positions, allowing it to effectively handle objects in different locations. Rotation helps improve the model’s robustness to image rotation, enabling it to adapt to variations in orientation. Simultaneously, mirroring contributes to the model learning features of symmetrical objects, thereby enhancing its generalization when dealing with symmetric entities. This is followed by a raised gray value operation, which extends the dynamic range of the image and makes the contrast between the light and dark areas of the image more pronounced. This helps the model to better capture detailed information in the image. In some cases, an image may appear too averaged due to insufficient lighting or other reasons. By increasing the grayscale value, the image can be made more vivid, avoiding over-averaging and helping the model to better understand the content of the image. Finally, the operation of adding Gaussian noise is performed. This noise is generated with a Gaussian distribution with zero as the mean, and its purpose is to introduce some randomness and uncertainty in the training data, so that the model can maintain its performance in the face of some noise and variations, without being overly affected by these perturbations. The operation of adding Gaussian noise introduces some distortions, including distorting high-frequency features and low-frequency components, but convolutional neural networks (CNNs) have the ability to learn to adapt to these distortions. After filtering the obtained images, the operations of cropping, flipping, mirroring, increasing the gray value, and adding Gaussian noise yielded 371, 199, 199, 769, and 769 images, respectively. As a result, we obtained 2307 cells images each with a size of 256 × 256 and used 80% of the dataset for training and the rest for testing.

## 3. Cells Segmentation Proposed Method

U-Net is a deep learning architecture designed for image segmentation tasks, and its name comes from the shape of the network structure, which presents a symmetrical U shape. This network structure is widely used in the field of image segmentation due to its unique design [41,42,43,44]. U-Net introduces jump connections and upsampling paths, which makes it better able to capture features at different scales and preserve the detailed information of the input image. The network architecture consists of two parts: an encoder and a decoder. The encoder consists of a convolutional layer and a pooling layer to capture high-level features in the input image and reduce the spatial resolution. Each convolution is followed by a downsampling step to reduce the size of the feature map. The decoder consists of an upsampling layer and a convolutional layer to upsample the features extracted from the encoder and connect them to the features of the corresponding encoder layer. Such a structure helps to introduce more upsampling details into the final segmentation output. At each layer of the decoder, the feature map of the corresponding encoder layer is connected to the feature map of the current decoder layer. These jump connections help to maintain more detailed information for better recovery of the segmented image. The output of U-Net is a segmentation map with the same dimensions as the input image, where each pixel corresponds to the predicted class of the pixel at the corresponding position in the input image. During the training process, optimization is usually carried out using, e.g., the cross entropy loss function.

U-Net++ is an extension and improvement of the traditional U-Net model [27]. It further improves the performance of the image segmentation task by introducing deeper hierarchies and richer feature fusion mechanisms. U-Net++ increases the depth of the overall network by introducing deeper sub-networks at each level of the encoder and decoder to better capture features at different scales and improve the expressive power of the model. In U-Net++, the single jump connection of traditional U-Net is replaced by dense jump connections. This means that each decoder layer is connected to all the corresponding layers of the encoder, not just the neighboring ones. This dense connection helps to utilize the information of the encoder layers more comprehensively and improves the efficiency of feature transfer. In addition, U-Net++ introduces a feature reweighting mechanism to reduce the weight of low-level features and make the network more focused on the contribution of higher-level features. This improvement increases the network’s focus on more important features, which helps to understand the image semantic information more accurately. Overall, U-Net++ significantly improves the performance of the image segmentation task by introducing more hierarchical structures and richer feature fusion mechanisms while retaining the simple but effective structure of U-Net.

### 3.1. SEAM-Unet++ Network

**Figure 3:**
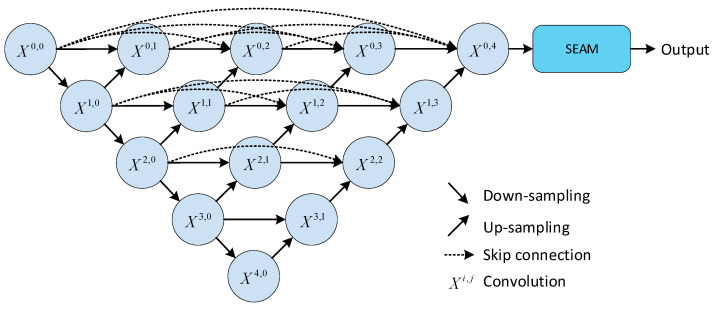
SEAM-Unet++ model.

The abovementioned method proves beneficial in processing images containing intricate details and sophisticated semantic structures; however, it falls short in producing satisfactory results when dealing with cell profile images exhibiting cells adhered together. To address this issue, it should be noted that the $X^{0,4}$ layer of Unet++ integrates all the features from preceding layers. To improve feature learning and representation, we direct its output to the SEAM module [45]. We avoid adding excessive attention mechanisms in other areas considering the risk of overfitting among other factors. Additionally, we limit the use of attention mechanisms in other areas to prevent overfitting and for other reasons. SEAM mitigates the deleterious effect of mutual occlusion among cells on segmentation outcomes. It accurately delimits occluded cell contours through increased attention towards these regions, thereby fortifying the robustness of the segmentation assignment. Furthermore, cell contour images are impacted by blurring and noise. The SEAM attention mechanism is able to notably decrease the interference of background noise on the segmentation results. This occurs as the model enhances the detailed information in the cell contour images, refines the segmentation detail and accuracy, and enables the model to acquire a better understanding and capture the minuscule features in the images. To illustrate, Figure 3 shows the architecture of SEAM-Unet++ as proposed in this study.

### 3.2. Attention Mechanism

In the field of computer vision, the attention mechanism is a key technique for modeling human perceptual processes. It enables a model to focus on task-relevant information while processing input data, thus improving model performance and efficiency [46,47,48,49]. A typical attention mechanism consists of the following steps: first, the input data (e.g., images) are transformed into high-level feature representations via a feature extraction network. Next, the attention mechanism calculates the attention weights for each feature, emphasizing the features that are helpful to the task. These weights are multiplied with the original features to produce a weighted feature representation. Eventually, these features processed by the attention mechanism are passed to the subsequent network for further task processing, such as classification, segmentation, etc. [50,51,52].

In practice, the attention mechanism plays an important role in several computer vision tasks. For example, for image categorization, it helps the model to focus more accurately on the category-related regions of the image. In target detection, the attention mechanism is used to determine the region of the image that contains the target. In image segmentation tasks, it can be used to guide the model to better capture the contours and details of the target object. In addition, the attention mechanism is widely used in image generation tasks to produce images with more detail and realism [53,54].

When observing daily life scenes, individuals tend to focus on distinctive areas and process them rapidly. For instance, when looking at a photograph, attentional mechanisms cause individuals to pay more attention to objects, people, or other compelling elements in the image, while ignoring details in the background. This attention enables individuals to quickly comprehend the scene, recognize objects, and respond adaptively to their surroundings. The above process can be formulated as (1).

(1) Attention=f(g(x),x)

The function *g*(*x*) represents the process of generating attention, which involves attending to the discriminated region. The function *f*(*g*(*x*), *x*) represents the processing of the input *x* based on the attention generated by *g*(*x*).

In an attention mechanism, there is usually a transformation representing the query (*Q*), key (*K*), and value (*V*), and the weights obtained using activation function. Given an input *x*, it can be represented as (2):

(2) Q,K,V=Linear(x)

This step converts the input *x* into the query *Q*, the key *K*, and the value *V* by means of a linear transformation (usually a fully connected layer), as shown in Formula (3).

(3) g(x)=σ(QK)

*σ* denotes the activation function, the number of commonly used activation functions such as Sigmoid and Softmax, etc., which are used to compute the attention weights and convert the inner product of the query and key to a probability distribution through the activation function. Then, the attentional weights g(x) to weight the value *V* are weighted to obtain the final attention output, as shown in Formula (4).

(4) f(g(x),x)=g(x)V

### 3.3. SEAM Attention Module

The SEAM module was initially proposed to address interclass occlusion’s problems that might cause alignment errors, local aliasing, and missing features. Initially, the SEAM module seeks to achieve multiscale features through different patches. These patches are small regions or sub-images that contain local information. They can be useful in identifying features, textures, edges, and more in the picture. A sequence of transformational operations is performed on a convolutional neural network. The initial operation applies a next depth separable convolution, utilizing residual concatenation to reduce parameter count and combat the issue of gradient vanishing. Following this, the outputs of convolutional layers with differing depths are combined through point-by-point convolution. The final operation utilizes two fully connected layers to fuse channel information. This enables the model to learn the relationship between unobstructed and occluded contours, compensating for any loss of data. Finally, the output of the fully connected layers undergoes processing with an exponential function, which extends the range from [0, 1] to [1, e], enhancing the results’ ability to tolerate positional errors. In the SEAM module, a simplified attention mechanism is used, which can be represented by the following equation:

The input *x* is linearly transformed to obtain the query *Q*, the key *K*, and the value *V*, as shown below.

(5) Q=LinearQ(x)

(6) K=LinearK(x)

(7) V=LinearV(x)

Here, *LinearQ*, *LinearK*, and *LinearV* denote the linear transformation of the input *x* to obtain the query *Q*, key *K*, and value *V*, respectively. Next, integrate global information, as shown in Formula (8):

(8) $$y_{i}=avg\_pool(Con_{i}(x))(i=0,1,2,3)$$

There are two steps in $Con_{i}$. First, a depth separable convolution is performed on x and the channels are divided into groups. The convolution operation is performed for each group so that each input channel can be processed separately, reducing the number of parameters. Then, the output of the depth-separable convolution is integrated across channels using point-by-point convolution. The *avg_pool* operation performs an average pooling operation on the output of $Con_{i}$. This is the process of downsampling the output feature map by averaging the values of each channel of the feature map to obtain a global message. The next step is to calculate the attention weights as shown in Formula (9):

(9) $$g(x)=Sigmoid(Linear_{fc}(Avg(y_{i})))(i=0,1,2,3)$$

Avg(y) denotes the average of the features obtained by taking different groups of depth-separable convolutional blocks. $Linear_{fc}$ performs a linear transformation on the averaged global information *y*. The purpose of this step is to learn, through training, how to map global information to another space in order to obtain the parameters needed for calculating attention weights. The sigmoid activation function is mainly useful in mapping the input to an output in the range (0, 1) as shown in Formula (10).

(10) $$Sigmoid(x)=\frac{1}{1+e^{-x}}$$

*Sigmoid* is a smooth, differentiable function that allows for efficient calculation of the gradient in backpropagation algorithms and thus model parameter updates. 

The obtained attention weights are subsequently weighted, as shown in Formula (11).

(11) $$y^{\prime }=g(x)⊙y$$

Here, the ⊙ symbol denotes the element-by-element multiplication. Finally, Formula (12) employs the natural exponential function to handle $y^{\prime }$.

(12) $$output=x⊙exp(y^{\prime })$$

Its purpose is to nonlinearly map and enhance the learned attention weights $y^{\prime }$, which are then used to weight the input *x*. This design allows the model to flexibly learn relationships in the input and dynamically adjust attention levels to different parts.

**Figure 4:**
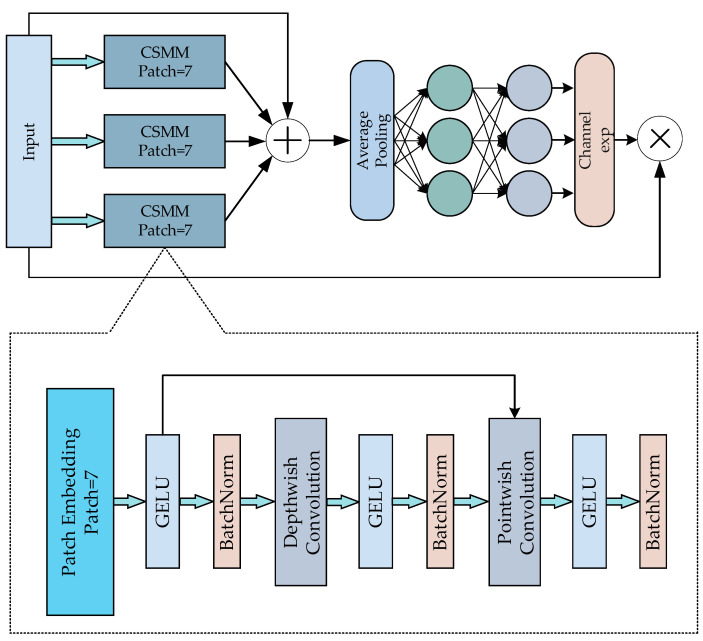
Illustration of SEAM. The top is the SEAM module, and the bottom is the CSMM (channel and spatial mixing module).

For the cell contour segmentation task, we opted to use a single type of patch since there is only one category involved. Based on our experiments, we discovered that the model offered optimal effectiveness in the segmentation of cell contours when the patch size was 7. To enhance anti-interference performance, we utilized three channel and spatial mixing modules (CSMMs). Every module was trained independently, using 7-patch sizes. This approach not only reduces the risk of overfitting, but also prevents high model complexity caused by additional convolutional kernels. To learn the correlation between the channel and spatial dimensions, we employed deeply separable convolution and validated its findings. The aforementioned measures enhance the precision of cell boundary delimitation when overlapping cells are present. The arrangement of SEAM is illustrated in Figure 4.

## 4. Evaluation Indexes

The relevant metrics for evaluating the neural network model’s effectiveness are precision, recall, IoU, and Dice. Binary classification problems classify samples into four types based on the combination of true and predicted categories: True Positive (TP), False Positive (FP), True Negative (TN), and False Negative (FN). Table 1 displays the confusion matrix of classification results.

Precision and recall are defined as Equations (13) and (14):

(13) $$Precision=\frac{TP}{TP+FP}$$

(14) $$Recall=\frac{TP}{TP+FN}$$

Accuracy is an intuitive and easy-to-understand metric. It simply represents the proportion of samples correctly classified by the model as a percentage of the total number of samples, which makes accuracy very intuitive in explaining and communicating model performance, as shown in Equation (15).

(15) $$\mathrm{Accuracy}=\frac{TP+TN}{TP+FP+FN+TN}$$

*IoU* is expressed as the ratio of the intersection area between the predicted bounding box $B_{prd}$ and the ground truth bounding box $B_{gt}$ to the union area of both bounding boxes, as shown in Equation (16).

(16) $$IoU=\frac{B_{gt}\cap B_{prd}}{B_{gt}\cup B_{prd}}$$

The *Dice* coefficient represents a means of determining the similarity of two sets, commonly utilized in image segmentation and related domains. This is calculated with the following specific Equation (17).

(17) $$Dice=\frac{2\times \left|A\cap B\right|}{\left|A\right|+\left|B\right|}$$

where *A* and *B* denote the binarized segmentation results and the ground truth of the model predictions; $\left|A\cap B\right|$ denotes the size of their intersection; $\left|A\right|$ and $\left|B\right|$ denote their sizes, i.e., the number of pixels, respectively. The *Dice* coefficient has a range of values between 0 and 1, where 1 indicates perfect overlap and 0 indicates no overlap. Consequently, the Dice metric is an efficient measure of similarities between two binary segmentation results.

## 5. Experiments

### 5.1. Experimental Configuration and Hyperparameter Settings

The hyperparameters of all models were optimized using a stochastic gradient descent (SGD) optimizer with an initial learning rate of 0.001, a momentum of 0.9, and a weight decay of 0.0001. The number of epochs was 200, and the batch size was 16. The loss function was chosen as BCEDiceLoss, which combines BCELoss (Binary Cross Entropy Loss) and Dice Loss. BCE Loss calculates the loss by comparing the difference between the output probabilities of the model and the true labels, while Dice Loss measures the loss by calculating the similarity between the predicted results and the true labels. The formula for BCEDiceLoss is usually a linearly weighted combination of BCE Loss and Dice Loss, and the weights for them in the experiments in this paper are chosen as 0.5 and 1, respectively. The input image size is 256 × 256 × 3. The experimental equipment configuration is shown in Table 2.

### 5.2. Comparison of the Performance of the Models

**Figure 5:**
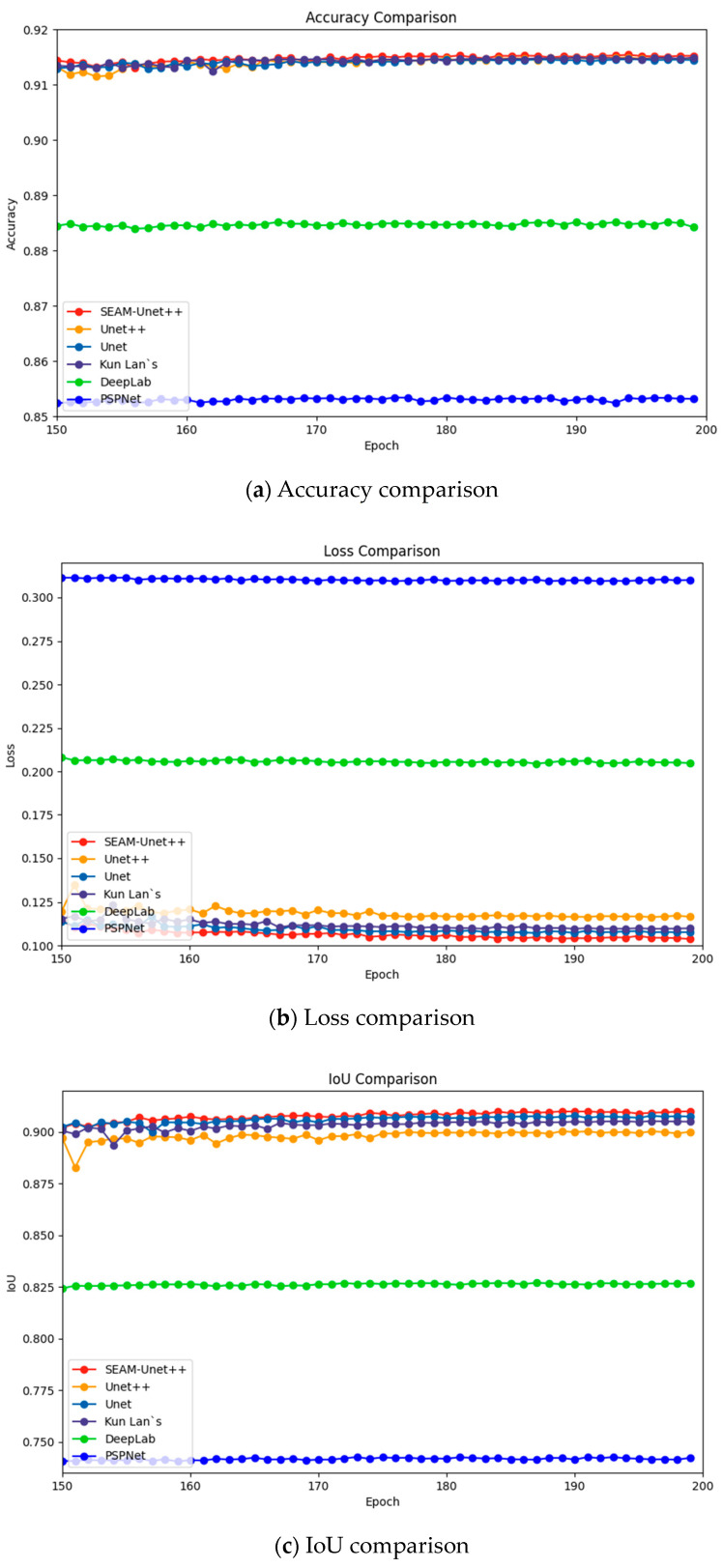

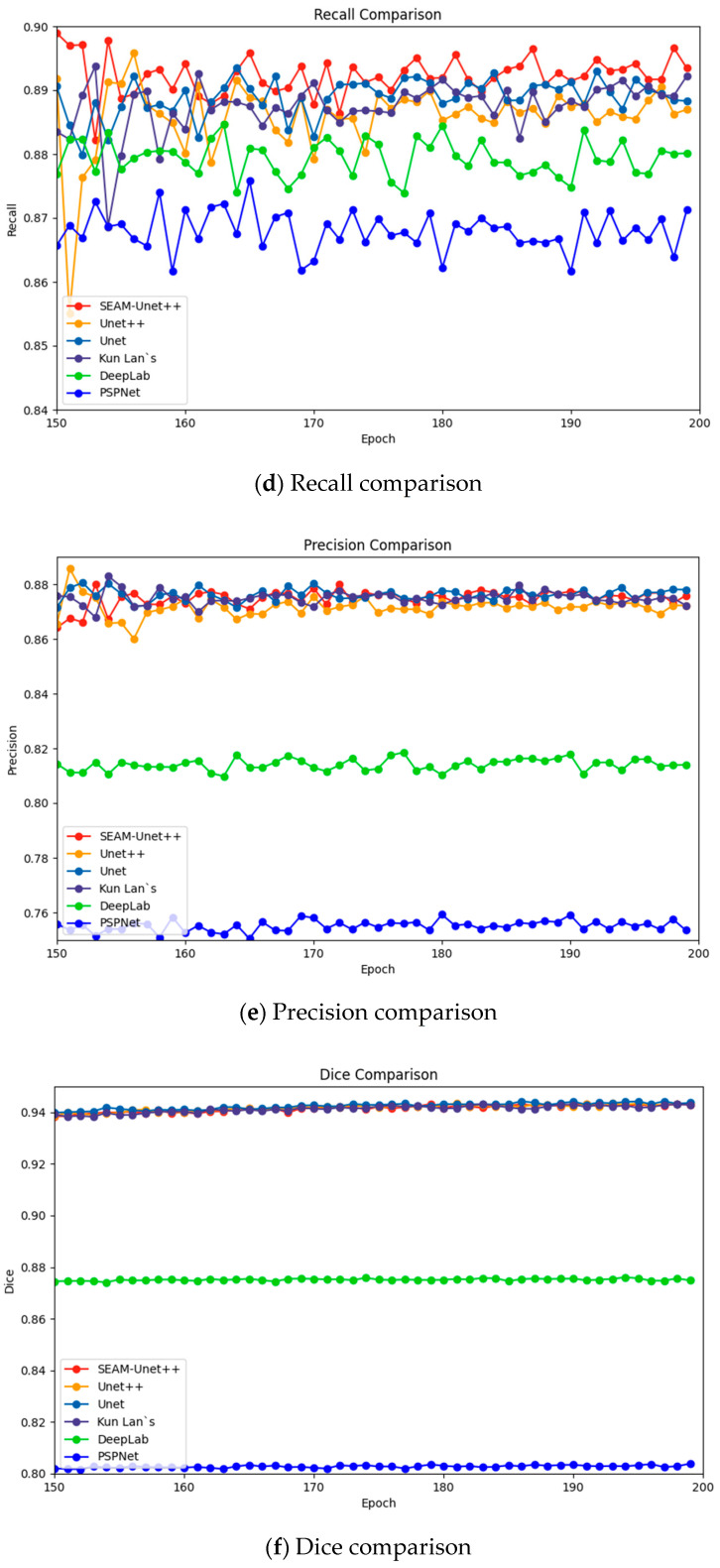
Parameter comparisons of different models.

In accordance with widely accepted validation guidelines [55,56], we compare SEAM-Unet++ and Unet++, Unet, PSPNet, DeepLab, and Kun Lan’s [57] model. PSPNet (Pyramid Scene Parsing Network) is a deep learning network specialized in scene parsing and semantic segmentation. The network is able to capture contextual information at different scales of the input image by introducing a pyramid pool structure. DeepLab is a series of deep learning models developed by Google that focus on solving the task of semantic segmentation of images. These models use deep differentiable convolution to reduce the number of parameters, incorporate spatial pyramid pooling to process images in a multiscale manner, and apply techniques such as inflated convolution and multiscale upsampling to improve the ability to accurately capture object boundaries and details. Kun Lan’s model and the one in this paper are both improvements on Unet++, and both are used to perform cell segmentation. The training parameters and configurations are as described in the previous section and the maximum results obtained after model convergence are recorded in Table 3 and Figure 5.

**Figure 6:**
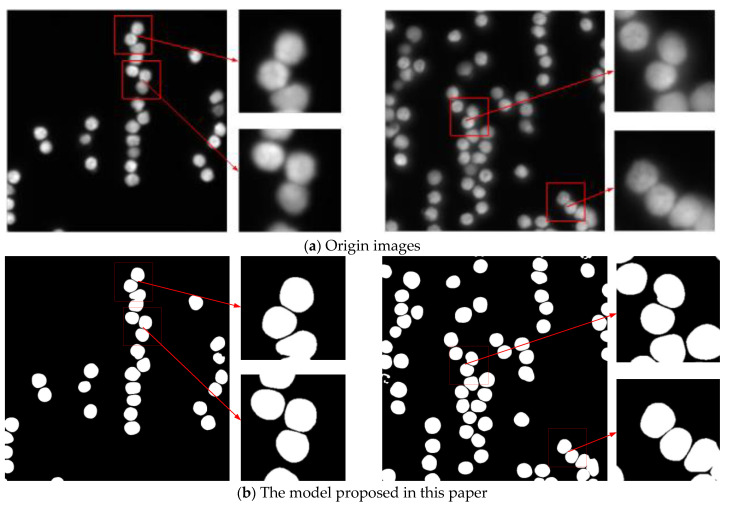

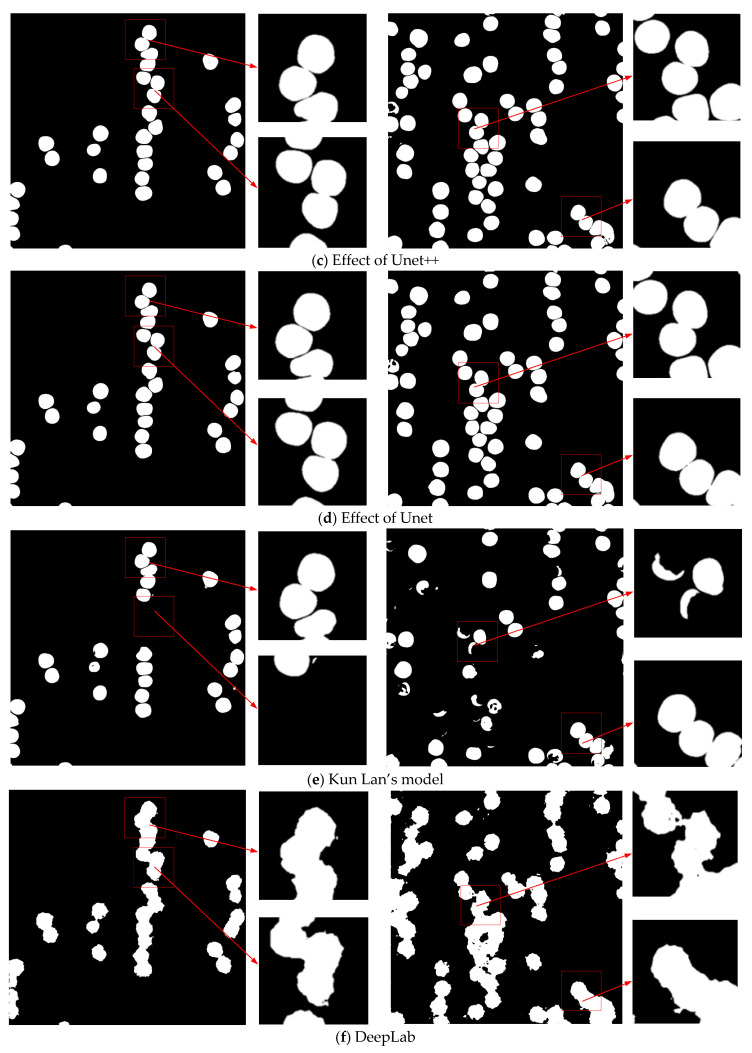

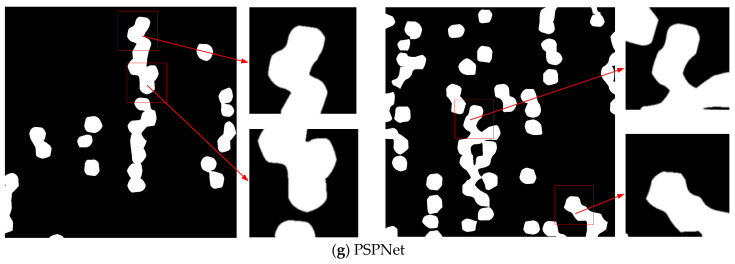
Comparison of segmentation effect of different models.

SEAM-Unet++ performs best in accuracy, proving that it successfully captures regions of the target object and accurately distinguishes the target from the background. Loss is used to measure the difference or error between the predicted output of the model and the ground truth. The lower the loss value, the closer the model’s predictions are to the actual labels and the better the model’s performance on the task. SEAM-Unet++ performs the best on the accuracy metrics with a minimum value of 0.1037, which further proves that the model in this paper successfully reduces the discrepancy between the predicted outputs and the ground truth during the training process. This is a strong indication that the model has achieved significant results in learning the key features in the data and is able to accurately capture the boundary, shape, and detail information of the target object. This strong performance supports the superiority of SEAM-Unet++ in image segmentation tasks, emphasizing the significant optimization achieved during the training process. IoU is the ratio of the intersection part of the model’s segmentation results to the connecting part of the ground truth. SEAM-Unet++ performs best in this metric at 0.9101, indicating that it is able to locate the target region more accurately. Recall is the ratio of the intersection part of the model’s segmentation results to the connecting part of the ground truth. Recall evaluates the ability of the model to not miss the positive examples during training. The performance of the model in this paper on this metric proves that it can effectively find the target object in the task, which is a reflection of the reliability of the model when facing real-world scenarios. Precision stands for the prediction accuracy of the positive examples, while Dice stands for the similarity between the prediction of the model in the segmentation task and the ground truth. SEAM-Unet++ achieves significant results in these two metrics, indicating that it has high accuracy and matching in labeling the target region. To further validate the ability of the model proposed in this paper to segment cell contours, we use the trained model to predict images outside the dataset, of which representative ones are shown in Figure 6.

It is important to note that accurately segmenting adherent cell contours is a crucial step in extracting individual cell contours, which directly impacts the determination of the cells’ nature. As shown above, the others all show varying degrees of contour sticking on the mask image when segmenting aggregated cells, while SEAM-Unet++ shows a significant improvement in the ability to completely segment the contours.

## 6. Conclusions

SEAM-Unet++ is a network structure that is created by combining the Unet++ network architecture and the SEAM attention mechanism. The SEAM module solves the issue of interclass occlusion to some extent, which can lead to alignment errors, local aliasing, and missing features. Considering that the final layer of Unet++ comprises the characteristics of all earlier layers, the output of this layer is given to SEAM. The aim is to enhance feature learning and representation, particularly with regard to cellular contour characteristics. A comparative experiment shows that SEAM-Unet++ is more effective than Unet++ and Unet for cell contour segmentation. Currently, there are many models for cell segmentation, but few models dedicated to FISH cell image segmentation. The method in this paper fills this gap to some extent. Although SEAM-Unet++ has a significant improvement in dealing with cell boundaries, it is undeniable that its performance in dealing with complex FISH images still needs to be improved.

Future work will entail expanding the applicability of existing models in the field of medical image processing beyond the confines of FISH images. This will involve exploring and enhancing the effectiveness of deep learning models on a wide spectrum of medical image types such as cardiac ultrasound and CT scan images, among others.

## Figures and Tables

**Table 1:** Confusion matrix of classification results.

Label	Predict	Confusion Matrix
Positive	Positive	TP
Positive	Negative	FN
Negative	Positive	FP
Negative	Negative	TP

**Table 2:** Experimental configuration.

Name	Parameter
CPU	Intel Xeon Gold 5318Y
GPU	NVIDIA A100
Programming language	Python 3.8.17
Deep learning framework	Pytorch 1.8.0

**Table 3:** Comparison of segmentation result data.

Model	Accuracy	Loss	IoU	Recall	Precision	Dice
SEAM-Unet++	0.9153	0.1037	0.9101	0.8977	0.8800	0.9432
Unet++	0.9149	0.1163	0.9003	0.8958	0.8856	0.9435
Unet	0.9146	0.1071	0.9078	0.8935	0.8805	0.9442
Kun Lan’s	0.9148	0.1095	0.9053	0.8937	0.8829	0.9432
DeepLab	0.8851	0.2043	0.8270	0.8847	0.8185	0.8960
PSPNet	0.8533	0.1095	0.9053	0.8937	0.8829	0.9432

## Data Availability

The dataset that supports the findings and conclusion of this study are available from the corresponding author upon reasonable request. The data are not publicly available due to privacy.
